# Molecular characterization and expression analysis of CSαβ defensin genes from the scorpion *Mesobuthus martensii*

**DOI:** 10.1042/BSR20171282

**Published:** 2017-12-15

**Authors:** Yange Lang, Xiaohuan Pi, Zhiyong Di, Qian Zhang, Huijuan Wang, Bingzheng Shen, Fangfang Li, Gaomin Liu, Yao Yu, Xuan Li, Yingliang Wu, Wenxin Li, Zhijian Cao

**Affiliations:** 1 State Key Laboratory of Virology, College of Life Sciences, Wuhan University, Wuhan 430072, China; 2 College of Basic Medicine, Lanzhou University, Lanzhou 730000, China; 3 College of Life Sciences, University of Science and Technology of China, Hefei 230022, China; 4 Department of Pharmacy, Renmin Hospital, Wuhan University, Hubei, 430060, China; 5 Key Laboratory of Synthetic Biology, Institute of Plant Physiology and Ecology, Shanghai Institute for Biological Sciences, Chinese Academy of Sciences, Shanghai 200031, China

**Keywords:** CSαβ defensin genes, constitutive transcription, expression pattern, inducible transcription, scorpion

## Abstract

Defensins are important components of innate host defence system against bacteria, fungi, parasites and viruses. Here, we predicted six potential defensin genes from the genome of the scorpion *Mesobuthus martensii* and then validated four genes from them via the combination of PCR and genomic sequence analysis. These four scorpion defensin genes share the same gene organization and structure of two exons and one phase-I intron with the GT-AG rule. Conserved motif and phylogenetic analysis showed that they belonged to the members of the invertebrate cysteine-stabilized α-helix/β-sheet motif defensin (CSαβ) defensin family. All these four CSαβ defensin genes have the expression feature of constitutive transcription (CON) by the whole scorpion infection model, promoter sequence analysis and dual luciferase assays. Further evolution and comparison analysis found that the invertebrate CSαβ defensin genes from most of arachnids and mollusks appear to share the expression pattern of CON, but those from insects and lower invertebrates (nematodes, annelids, cnidarians and sponges) seem to have identical inducible transcription (IND) after being challenged by microorganisms. Together, we identified four scorpion CSαβ defensin genes with the expression feature of CON, and characterized the diversified expression patterns of the invertebrate CSαβ defensin genes, which will shed insights into the evolution of the invertebrate CSαβ defensin genes and their expression patterns.

## Introduction

Defensins are small cationic antimicrobial peptides containing three or four intramolecular disulphide bonds formed by six or eight cysteine residues in a complex folded arrangement of two or three antiparallel β-sheets with or without an α-helix structure [1–4]. Defensins are important components of the host immune system and produced by a wide range of organisms including vertebrates, invertebrates, plants and fungi, which have defensive functions against a broad array of infectious pathogens like bacteria, fungi, parasites and viruses [3,5,6]. Generally, the spatial structures of defensins differ according to the arrangement of conserved cysteine residues [1–3,7].

Vertebrates’ defensins have six cysteine residues and are divided into three distinct families: α, β and θ defensins, which have lengths of 29–35, 35–45 and 18 amino acid residues respectively [8]. Defensins have ubiquitously been identified among invertebrates, predominantly in mollusks [9,10], nematodes [11–13] and arthropods [14]. The classification of invertebrate defensins differs based on the complex criteria [3]. Invertebrate defensins can primarily be classified into two classes. One class is the invertebrate cysteine-stabilized α-helix/β-sheet motif defensin (CSαβ defensin), containing an α-helix linked to an antiparallel two-stranded β-sheet by disulphide bridges and is found in mollusks, nematodes and arthropods [15,16]. The other class is the invertebrate big defensin, which has a disulphide array stabilized β-sheet structure different from the invertebrate CSαβ motif [17,18] and exists mainly in mollusks [19] but also in arthropods [20].

Scorpions are one of the most ancient terrestrial venomous animal lineages [21]. Scorpions have more than 400 million years of evolutionary time, and so are considered to be living fossils [22]. Scorpions have a strong ability to adapt to different environments and are widely distributed on all continents except Antarctica [23]. More than 2329 scorpion species classified into 17 families have been recorded to date (http://www.ntnu.no/ub/scorpion-files/specieslist.php). The scorpion *M. martensii* belonged to the family Buthidae and was used to be an important raw material in Chinese Traditional Medicine for the treatment of some nervous system diseases [24].

As one kind of arachnid animals, scorpions mainly rely on innate immune system against infectious microorganisms like insects [25]. Especially, defensins are a class of ubiquitously expressed cationic antimicrobial peptides (CAPs) that play an important role in innate defence [26]. Scorpion defensin research can be traced back as early as 1993, when Cociancich et al. [27] isolated and structurally characterized a defensin from the scorpion *Leiurus quinquestriatus*. In 1996, Ehret-Sabatier et al. [28] identified *Androctonus* defensin with three disulphide bridges from the haemolymph of the scorpion *Androctonus australis*. Insect defensins are considered to be a group of inducible small-sized antibacterial peptides [29], but chelicerate defensins are implicated to have complicated alternative defensive systems: up-regulation dependent on inducible (IND) or constitutive transcription (CON) and constitutive production [30]. Scorpion is one of chelicerate animals, but only one scorpion defensin Cll-dlp was demonstrated to accumulate in the haemolymph in response to septic injury, independent of transcriptional regulation [30]. Expressive characteristics of defensin genes from scorpions and invertebrates still need to be systematically studied and analysed, which provides insights into the evolution of defensins and innate immune system.

Our group previously determined the draft-genome sequence of the scorpion *M. martensii* (Scorpions: Buthidae) [31]. In the present work, we predicted six potential defensin genes from the scorpion *M. martensii* genome, and then experimentally validated four defensin genes. Gene organization and evolutionary analyses confirmed that they are the members of the invertebrate CSαβ defensin gene family. Their expression features were identified by an intact scorpion infection challenge, promoter sequence analysis and dual luciferase assay. Finally, we summarized and compared the expression patterns of almost all CSαβ defensins from the kingdom of invertebrates.

## Materials and methods

### Annotation of defensin genes

The NCBI database (http://www.ncbi.nlm.nih.gov/protein/) was used as a search engine for nucleotide sequences of scorpion defensins. Then, the downloaded nucleotide sequences of scorpion defensins were used to search in the scorpion *M. martensii* genome (GenBank number BioProject PRJNA171479) by BLAST as previously reported [31] and obtained several candidate defensin genes. These predicated defensin genes were further confirmed in the transcriptome of the scorpion *M. martensii* followed by ORF forecasting using a NCBI software (http://www.ncbi.nlm.nih.gov/gorf/orfig.cgi) to find the corresponding proteins. The signal peptides of the corresponding proteins were predicted by SignalP3.0 (http://www.cbs.dtu.dk/services/SignalP/). Multiple alignments were achieved by GeneDoc.

### Preparation of genomic DNA and PCR amplification

Genomic DNA was isolated as previously described [31]. Simply intact individuals of the scorpion *M. martensii* were washed three times with 75% ethanol and grounded to fine powder in liquid nitrogen. Then genomic DNA extraction was performed using TIANamp Genomic DNA Kit (Tiangen, China) according to the manufacturer’s instructions.

Primers (Supplementary Table S1) from 5′-UTR and 3′-UTR regions of the predicted defensin genes from the scorpion *M. martensii* genome were picked up to amplify the corresponding genomic DNA by PCR. Amplification was performed with one cycle of 5 min at 95°C, 30 cycles of 40 s at 95°C, 40 s at 58°C, 150 s at 72°C, and a final cycle of 10 min at 72°C using Ex Taq (TaKaRa, China). PCR products were purified using the DNA Clean-up Kit (CWBio, China) and ligated to pGEM-T Easy Vector (TaKaRa, China). Sequencing was performed by Tsingke Biological Technology.

### Phylogenetic analysis

Mature peptide sequences of defensins from the scorpion *M. martensii* and the other species including vertebrates and invertebrates were aligned by ClustalX 1.83. Phylogenetic tree of defensin superfamily was constructed using Neighbor-Joining method in MEGA 5.1 (http://www.megasoftware.net). Bootstrap sampling was reiterated 10000 times. The detailed sequence information for the analysis of defensins is in Supplementary Table S2.

### Preparation of bacterial materials

The bacterial strains *Escherichia coli* (AB94012) and *Staphylococcus aureus* (AB94004) were purchased from China Center for Type Culture Collection (CCTCC). The bacterial materials were acquired as following. Bacteria cultured in LB medium to OD_600_ =0.6 at 37°C was collected by centrifuging for 3 min at 6000 rpm. Then the collections were suspended in PBS for intact scorpion challenge. For cell stimulation, the supernatant was further smashed by ultrasonic and cleaned through a 0.22-μm filter. The filtrate was quantified by Pierce BCA Protein Assay Kit (Thermo Scientific, U.S.A.) according to the manufacturer’s instructions and stored at −20°C at a final concentration of 50 mg/ml.

### Intact scorpion infection model and real-time PCR analysis

The scorpion *M. martensii* individuals were challenged according to the modified protocol as previously described [30,32,33]. Simply, adult male *M. martensii* were maintained in the laboratory with sufficient water and food for 2 weeks before experimentation. Individuals of the test group were pricked with a fine needle with 5 μl of 200 ng/ml lipopolysaccharides (LPS, Sigma), 500 ng/ml lipoteichoic acid (LTA, Sigma), 50 μg/ml *E. coli* or 50 μg/ml *S. aureus* by a puncture between the second and third segments of the scorpion metasoma. Individuals of the control group were injected with 5 μl PBS; 36 h post injection, scorpions were washed for RNA extraction followed by cDNA synthesis and real-time PCR assay as previously described [31]. cDNA was synthesized using the First Strand cDNA Synthesis Kit (Thermo Scientific, U.S.A.). Real-time PCR was performed using the SYBR Green PCR assay and an ABI 7500 system according to the manufacturer’s instructions. The relative mRNA expression of four validated defensin genes from the scorpion *M. martensii* was normalized to β-actin. Primers used in the real-time PCR were summarized in Supplementary Table S3.

### Cloning, sequencing and analysis of promoter regions

According to the whole genome sequence of the scorpion *M. martensii*, gene-specific primers (Supplementary Table S4), located at ~1–2 thousand nucleotides upstream the translation start site of the validated four scorpion defensin genes, was used to clone their corresponding potential promoter regions. Amplification was performed with one cycle of 5 min at 95°C, 30 cycles of 40 s at 95°C, 40 s at 55°C, 90 s at 72°C, and a final cycle of 10 min at 72°C using ExTaq. PCR products were then purified, cloned and sequenced as above. Potential transcription factor binding sites upstream the initiation codon were searched using the P-Match program (http://www.generegulation.com/pub/programs.html#pmatch) and the AliBaba2 program (http://www.cs.uni-magdeburg.de/grabe/alibaba2) against the TRANSFAC database.

### Cell culture and dual luciferase assay of promoter activity

Human embryonic kidney (HEK293T) cells and *Drosophila* S2 cells were obtained from CCTCC. HEK293T cells were cultured in Dulbecco’s modified Eagle’s medium (Gibco/Life Technologies, U.S.A.) supplemented with 10% FBS (Gibco/Life Technologies, U.S.A.) and 1% penicillin/streptomycin in a humidified 5% CO_2_ incubator at 37°C. *Drosophila* S2 cells were cultured in Schneider’s Insect medium (Sigma, U.S.A.), supplemented with 10% FBS and 1% penicillin/streptomycin at 28°C.

The firefly luciferase reporter vectors were constructed by using the recognition sequence of MluI and XhoI to insert the cloned promoter region into pGL3 basic promoter vector. Primers used for dual luciferase analysis are described in Supplementary Table S5. HERK293T or *Drosophila* S2 cells were seeded in 24-well plates 24 h before transfection. And then 1 μg constructed firefly luciferase reporter vector and 50 ng *Renilla* luciferase plasmid pRL-TK (Promega, U.S.A.) as an internal control were transfected using Lipofectamine 2000 Transfection Reagent (Invitrogen, U.S.A.) following the manufacturer’s instructions. Twenty-four hours post transfection, cells were stimulated with bacterial materials *E. coli* (50 μg/ml), *S. aureus* (50 μg/ml), LPS (200 ng/ml) and LTA (500 ng/ml) respectively. Cells were assayed 48 h later with Dual Luciferase Assay (Promega, U.S.A.) according to the manufacturer’s instructions.

### Statistical analysis

All assays were performed in triplicate. Data from repeated experiments are expressed as the means ± S.D.

## Results

### Annotation of defensin genes in the scorpion *M. martensii* genome

**Figure 1:**
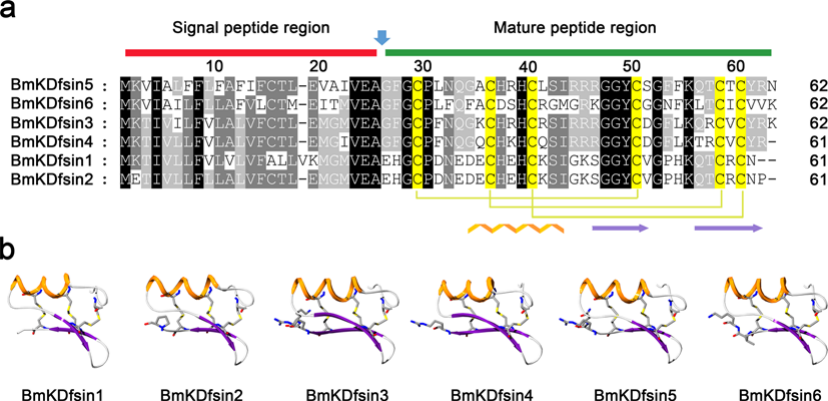
Six potential defensins annotated from the genome of the scorpion *M. martensii* (**a**) Multiple sequence alignment of six potential defensins from the scorpion *M. martensii* genome. The red line indicates the deduced signal peptide regions of the defensins, and the green line shows the deduced mature peptide regions of the defensins. The six conserved cysteine residues are highlighted with yellow background and form three pairs of intramolecular disulphide bridges that induce the CSαβ fold. (**b**) The predicted 3D structures of the six potential defensins from the scorpion *M. martensii*. Their 3D structures were modelled using the arthropod defensin (PDB code: 2ru0) as a template by the SWISS-MODEL server (http://swissmodel.expasy.org/). α-helical and β-parallel regions are highlighted with orange and purple colours respectively.

Defensins from scorpion species were first identified in *L. quinquestriatus*, followed by *A. australis* [27,28]. A total of 14 scorpion defensins were retrieved from online databases. A scorpion defensin query set was constructed and used in a tblastn search against the *M. martensii* genome. E-values less than 10^−4^ were used as a cut-off for significant hits of candidate defensin genes. Each hit was further verified manually based on similarities in sequence, cysteine patterns and gene structural organization. Six defensin genes were annotated in the scorpion *M. martensii* genome and named as BmKDfsin1, BmKDfsin2, BmKDfsin3, BmKDfsin4, BmKDfsin5 and BmKDfsin6 (Supplementary Table S6) respectively. These six defensin precursors consist of 61 or 62 amino acid residues, including 24 or 25 signal peptide residues and 37 or 38 mature peptide residues (Figure 1a), which is consistent with previously reported lengths of defensin precursors and mature peptides [34,35]. Each of these six defensins has six conserved cysteine residues which probably form three pairs of intramolecular disulphide bridges to induce the CSαβ fold. Their 3D structures were modelled using the arthropod defensin as a template (PDB code: 2ru0) on the SWISS-MODEL server (http://swissmodel.expasy.org/). The 3D structures display that six putative defensins from the scorpion *M. martensii* are composed of an α-helix linked to an antiparallel two-stranded β-sheet by three disulphide bridges (Figure 1b), suggesting that they all belong to the members of the invertebrate CSαβ defensin family.

### Validation of defensin genes from the genome of the scorpion *M. martensii*

**Figure 2:**
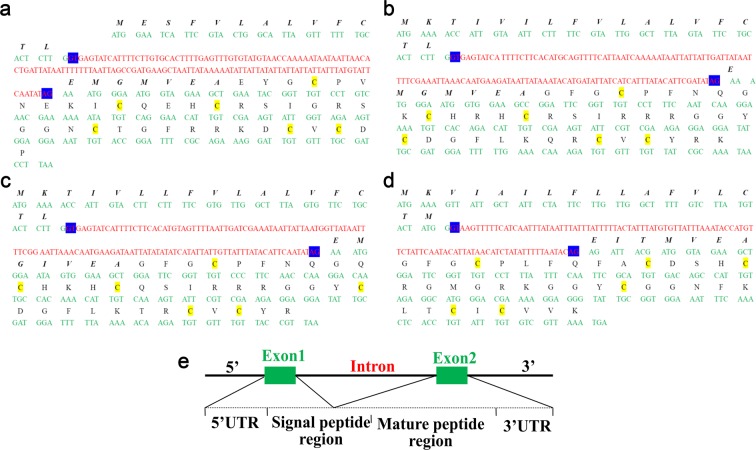
Sequence analysis of the four defensin genes validated from the scorpion *M. martensii* (**a**) BmKDfsin2. (**b**) BmKDfsin3. (**c**) BmKDfsin4. (**d**) BmKDfsin6. (**e**) Gene organization and structure analysis of four defensins from the scorpion *M. martensii*. Green letters represent exon sequences, and red letters are intron sequences. GT-AG splicing sites are shaded with blue background. The letters above the nucleotides are the corresponding amino acid sequences, the signal peptide regions are indicated by italics and bold letters, and the mature peptide regions are indicated with black letters. Cysteine residues are highlighted with yellow background.

BioProject PRJNA171479 was a draft genome sequence of the scorpion *M. martensii*. To validate whether the six newly discovered putative defensin genes truly existed in the genome of the scorpion *M. martensii*, PCR using 5′-UTR and 3′-UTR primers was performed and five amplified products were cloned. Four of these five PCR products were close to their predicted lengths (1449 bp for BmKDfsin2, 1492 bp for BmKDfsin3, 1265 bp for BmKDfsin4 and 991 bp for BmKDfsin6), while the 600-bp fragment for BmKDfsin1 was much shorter than the predicted size (945 bp) and no fragment for BmKDfsin5 was acquired (Supplementary Figure S1). Unfortunately, although we tried our best to design several pairs of primers and amplify, we still failed to get the predicated genomic fragments of BmKDfsin1 and BmKDfsin5. We thought that the existence of some wrong sequence information in the current draft genome version of *M. martensii* could account for our failure to achieve genomic sequences of BmKDfsin1 and BmKDfsin5. Finally, sequencing analysis of four DNA fragments with right lengths showed identical amino acid coding sequences to their counterparts annotated from the *M. martensii* genome (Figures 1a and 2a–d). Thus, we confirmed the existence of four CSαβ defensin genes (*BmKDfsin2, BmKDfsin3, BmKDfsin4* and *BmKDfsin6*) in the scorpion *M. martensii* genome.

### Genomic organization and classification of defensin genes from the scorpion *M. martensii*

Based on PCR validated sequence information, the gene organizations and structures of BmKDfsin2, BmKDfsin3, BmKDfsin4 and BmKDfsin6 were further analysed. These four scorpion CSαβ defensins share identical gene organization of two exons and one intron, where the first exon encodes the 5′-UTR region and part of the signal peptide, whereas the other exon encodes the remainder of the signal peptide, the mature peptide and the 3′-UTR region (Figure 2a–d). The intron has a consensus splice junction following the GT-AG rule [36]. Moreover, the gene organization of the four putative defensins closely resembles other mollusk and arthropod defensin genes: (i) the exon encoding the mature defensin is not split by any intron and (ii) the intron flanking the exon encoding the mature defensin shares a strict level of phase conservation (phase I) (Figure 2a-e). The mature defensin encoded by the second exon likely displays folding autonomy as one structural entity [35,37]. These four putative defensins also contain six cysteine residues in a CX_4–15_CX_2_HCX_6–9_GX_1_CX_4–9_CX_1_C distribution, the typical cysteine pattern of the invertebrate CSαβ defensins that induces the CSαβ fold [35,36]. These features further suggest that the four PCR-validated defensin genes from the scorpion *M. martensii* belong to the invertebrate CSαβ defensin family.

**Figure 3:**
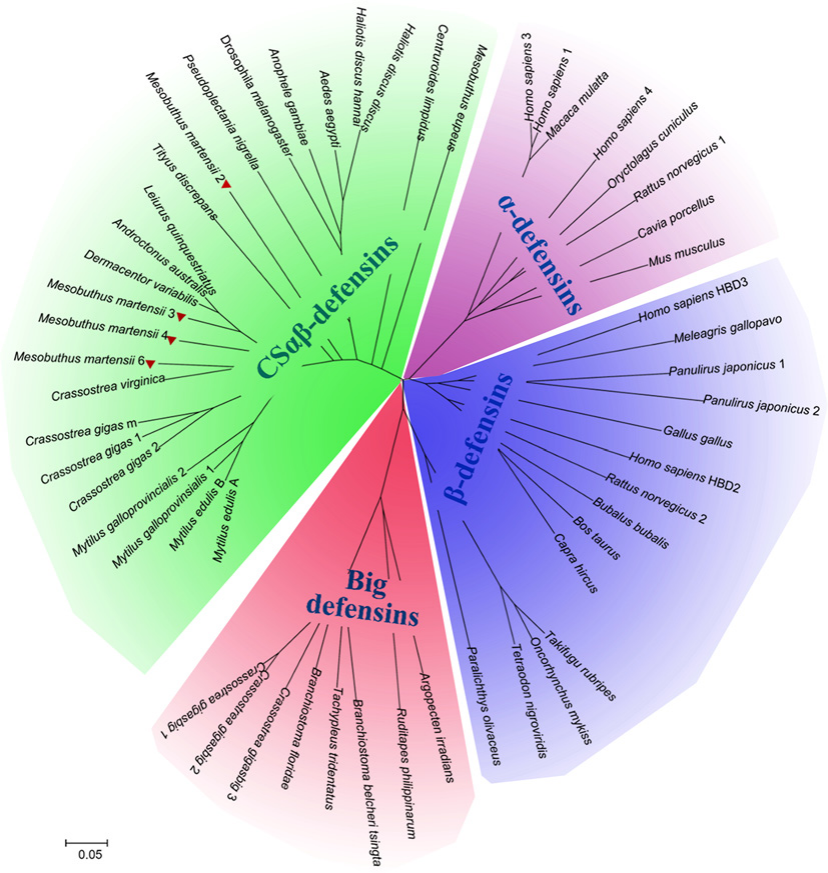
Phylogenetic analysis and classification of defensin genes from the scorpion *M. martensii* The tree was constructed using the Neighbor-Joining method in MEGA 5.1. Bootstrap sampling was reiterated 10000 times. We obtained a similar topology tree with the Maximum-Parsimony method in MEGA 5.1. The sequence information used in the defensin analysis is included in the Supplementary Table S2. The phylogenetic tree showed that the animal defensin superfamily clusters into four families: CSαβ-defensins, big defensins, α-defensins and β-defensins. The four defensins from the scorpion *M. martensii* are highlighted with red triangles and clustered into the invertebrate CSαβ defensin family clade only.

After confirming that their gene organizations and distribution of cysteine residues were consistent with those of the other invertebrate CSαβ defensins, we further estimated the evolutionary status of these four putative scorpion defensins by building a phylogenetic tree including sequences from other defensin families belonging to a variety of species and phyla. Phylogenetic analysis of vertebrate α- and β-defensin, invertebrate CSαβ defensin, and big defensin sequences revealed that the four PCR-validated putative defensins (BmKDfsin2, BmKDfsin3, BmKDfsin4 and BmKDfsin6) from the scorpion *M. martensii* were clustered into the invertebrate CSαβ defensin family (Figure 3).

### CON of CSαβ defensin genes from the scorpion *M. martensii*

**Figure 4:**
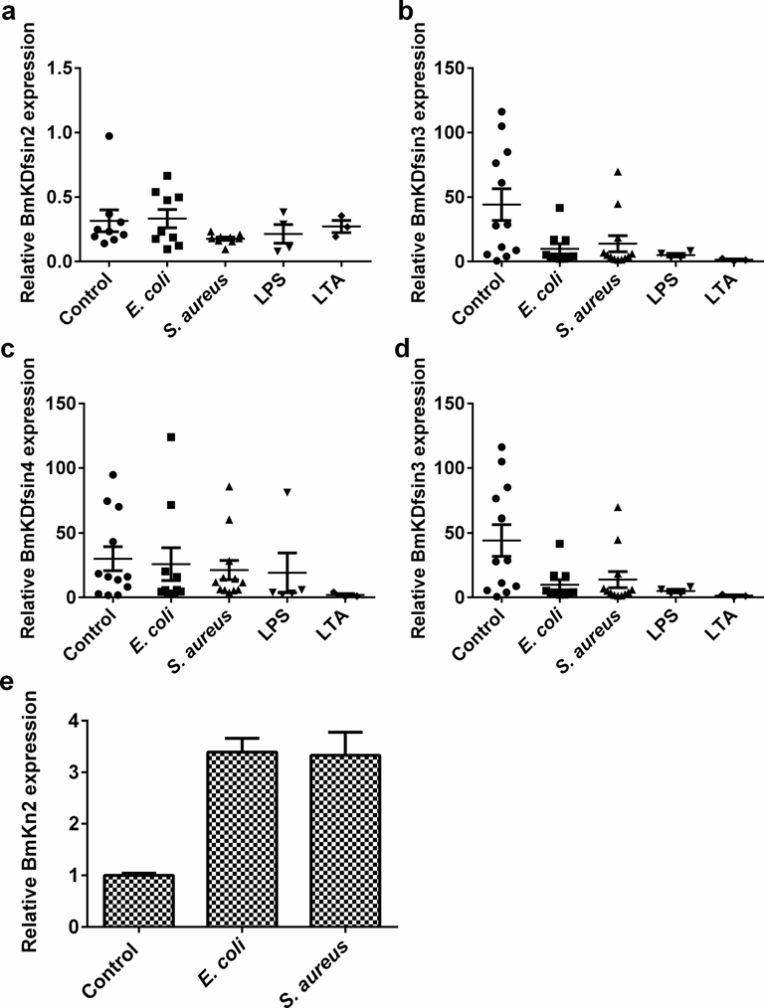
*In vivo* transcriptional activity analysis of four defensins from the scorpion *M. martensii* The mRNA expression of four defensins (BmKDfsin2, BmKDfsin3, BmKDfsin4 and BmKDfsin6) from the scorpion *M. martensii* was analysed. The scorpion *M. martensii* was challenged with 5 μl of *S. aureus* (OD_600_ =0.6), *E. coli* (OD_600_ =0.6), LPS (200 ng/ml) or LTA (200 ng/ml). PBS was used as an unchallenged control. After 36 h, the whole scorpions were ground and *defensin* mRNA expression was analysed. The antibacterial peptide gene *BmKn2* was detected as the positive control. (**a**) BmKDfsin2. (**b**) BmKDfsin3. (**c**) BmKDfsin4. (**d**) BmKDfsin6. (**e**) BmKn2.

Defensins are important components of host innate immune systems with defensive function against pathogens. The transcription of defensin gene is either inducible or constitutive when challenged by a pathogenic microorganism. We developed an intact scorpion (*M. martensii*) challenge model using *E. coli, S. aureus*, LPS or LTA to determine whether the four scorpion defensin genes (*BmKDfsin2, BmKDfsin3, BmKDfsin4* and *BmKDfsin6*) undergo inducible or constitutive mRNA expression *in vivo*. Scorpions were injected with bacteria or bacterial components. After 36 h, the scorpions were ground to analyse the mRNA expression of the four scorpion defensin genes. Real-time PCR indicated no obvious difference of their mRNA expression between the treated and untreated groups (Figure 4a–d). However, we found that some genes encoding antibacterial peptides like BmKn2 from the scorpion *M. martensii* can be induced using the same sample (Figure 4e). This solidly proved the validity of our intact scorpion challenge model. Thus, BmKDfsin2, BmKDfsin3, BmKDfsin4 and BmKDfsin6 from the scorpion *M. martensii* were revealed to constitutively express at the transcriptional level.

**Figure 5:**
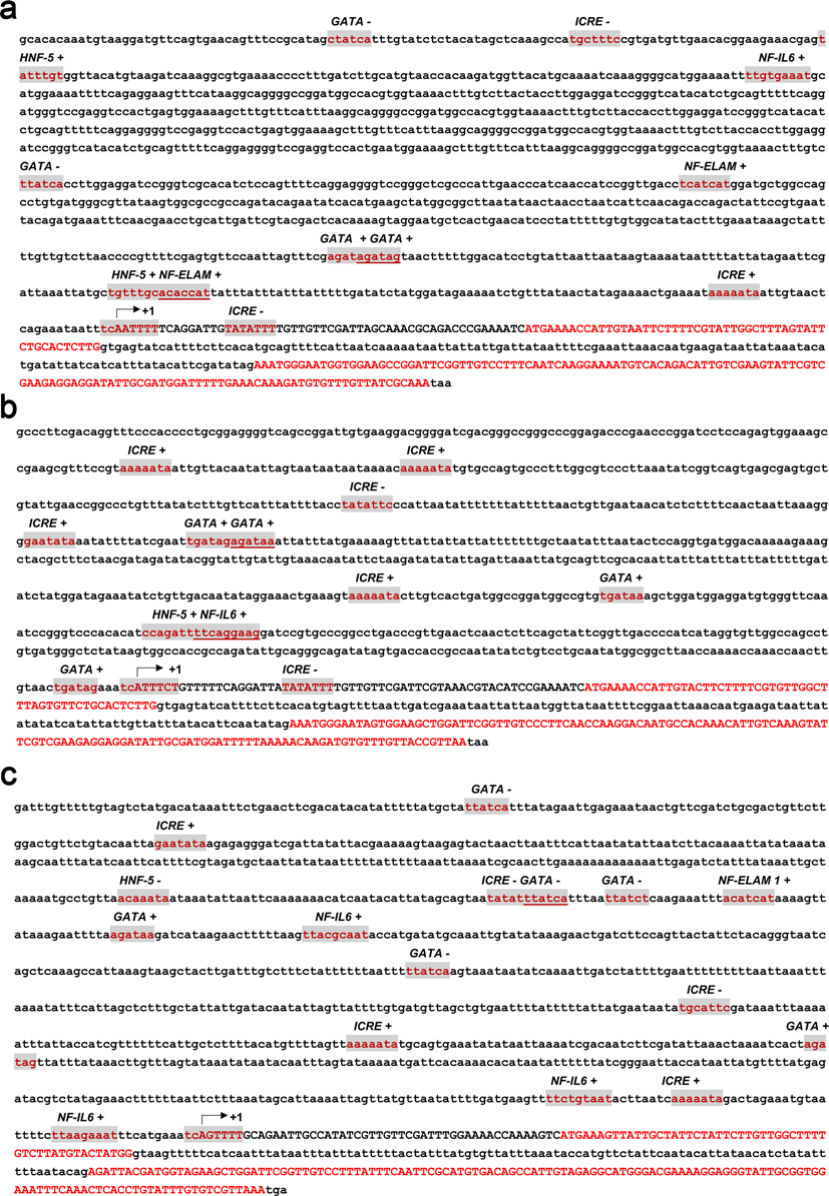
Promoter sequence analysis of three defensin genes from the scorpion *M. martensii* The cloned promoter regions of BmKDfsin3, BmKDfsin4 and BmKDfsin6 were sequenced and analysed to identify potential transcriptional regulating elements. The potential transcriptional regulating elements are highlighted with red letters and grey background. The sequences of the ORFs are presented in red capital letters. (**a**) BmKDfsin3. (**b**) BmKDfsin4. (**c**) BmKDfsin6.

Constitutive mRNA expression of the four scorpion defensins observed in the challenge experiment suggests a lack of immune response elements in the upstream regulatory regions of their promoters. Consequently, we attempted to clone and characterize their promoter regions based on the sequence of the scorpion *M. martensii* genome. Among the PCR-validated four scorpion defensins, the putative promoter regions of only three defensin genes (*BmKDfsin3, BmKDfsin4* and *BmKDfsin6*) were successfully amplified. They all covered ~1500 nts upstream of the initiation codon (Supplementary Figure S2). We tried hard and designed several pairs of primers and attempted a number of PCR methods but we failed to successfully amplify the promoter sequence of BmKDfsin2. We attributed it to that the the current assembled genome of the scorpion *M. martensii* that it is a draft genome and the upstream promoter sequence of BmKDfsin2 is probably wrongly assembled. Those three promoter regions were sequenced and first analysed to detect potential initiator elements according to the eukaryotic initiator element consensus [(TC)CA^+1^N(TA)(TC)(TC)(TC)] (N could be any of the four bases and A^+1^ is the first base of the transcription start site) [33,36]. BmKDfsin3, BmKDfsin4 and BmKDfsin6 have initiator elements with conserved sequences of TCA^+1^ATTTT, TCA^+1^TTTCT and TCA^+1^GTTTT respectively (Figure 5). The initiator element is an alternative promoter element capable of replacing the function of the TATA box, and its presence potentially explains the absence of a TATA box ~30 nts upstream of the transcription start site [38]. Next, the P-Match program and AliBaba2 program were used in conjunction with the TRANSFAC database to identify other putative transcription factor binding sites in the promoter regions of BmKDfsin3, BmKDfsin4 and BmKDfsin6 [39,33]. The result showed that their promoter regions have a variety of transcription factor binding sites, including nuclear factor interleukin 6 (NF-IL6) consensus TKNNGNAAK (K is G or C), GATA factor consensus WGATAR (W is A or T, and R is A or G), interferon consensus response element (ICRE) motif RAAWRYA (Y is C or T), hepatic nuclear factor 5 (HNF-5) consensus TRTTTGY, and nuclear factor endothelial leucocyte adhesion molecule 1 (NF-ELAM1) element WCAKCAK (Figure 5). Although the number of transcription factor binding sites varies between BmKDfsin3, BmKDfsin4 and BmKDfsin6, the types of response elements are similar. Briefly, BmKDfsin3 promoter region contains one NF-IL6, four GATA factor, three ICRE, one HNF-5 and two NF-ELAM1 sites (Figure 5a). BmKDfsin4 promoter region has four copies of GATA factor, one copy of NF-IL6, six copies of ICRE, and one copy of HNF-5, but no copy of NF-ELAM1 (Figure 5b). The promoter region of BmKDfsin6 covers six copies of GATA factor motif, two copies of NF-IL6 element, five copies of ICRE, one copy of HNF-5 consensus and one copy of NF-ELAM1 site (Figure 5c). All these elements are homologous to transcription factor binding sequences previously characterized in *Drosophila* [40], mosquitoes [39] and scorpions [33,36]. Thus, these conserved elements likely play similar roles in the regulation of *BmKDfsin3, BmKDfsin4* and *BmKDfsin6* genes during immune response. Their lack of the nuclear factor κB (NF-κB) binding site, which is a critical member of inducible immune factors and has been found in promoter regions of almost inducible *SCAMP* genes in animals [41], suggests that BmKDfsin3, BmKDfsin4 and BmKDfsin6 have the expression feature of CON.

**Figure 6.**
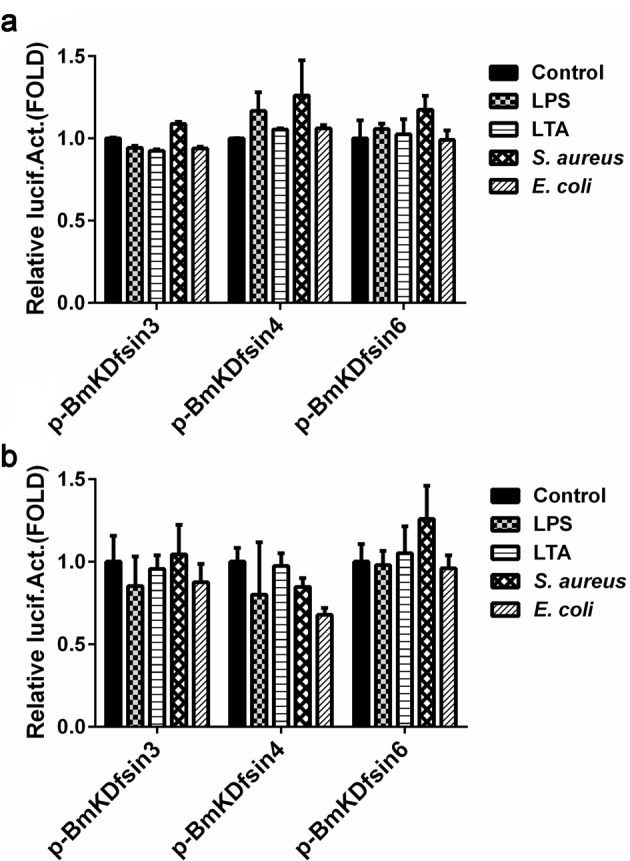
Dual luciferase assay of the promoter activity of three defensin genes in HEK293T and *Drosophila* S2 cells HEK293T or *Drosophila* S2 cells were transfected with pBmKDfsin-3, 4 or 6 vector respectively. At 6 h post transfection, the cells were treated with 200 ng/ml LPS, 500 ng/ml LTA, 50 μg/ml *S. aureus* or *E. coli* bacterial materials. PBS treatment was used as the negative control. The dual luciferase assay was performed at 48 h post transfection. (**a**) HEK293T cell line. (**b**) *Drosophila* S2 cell line.

Complementary to the promoter sequence analysis, their promoter activities were measured by a luciferase assay. Upstream promoter regions of BmKDfsin3, BmKDfsin4 and BmKDfsin6 were subcloned into luciferase reporter vectors called pBmKDfsin-3, 4 and 6 respectively. Because there is no cultured scorpion cell lines to date, the constructs were transfected into the human HEK293T cells which have high transfection efficiency or the S2 cells from *Drosophila* which have closer relationship with scorpions than humans. Their promoter activities, as indicated by the ratio of firefly luciferase and *Renilla* luciferase activities, did not change upon stimulation with bacterial components (Figure 6). Taken together, the result was consistent with their *in vivo* mRNA expression and promoter sequence characters, which firmly concluded that BmKDfsin3, BmKDfsin4 and BmKDfsin6 are constitutive at the transcriptional level.

## Discussion

### CON of defensins or defensin-like genes from scorpions

We validated four defensin genes (*BmKDfsin2, BmKDfsin3, BmKDfsin4* and *BmKDfsin6*) from the scorpion *M. martensii* at both the genome and cDNA levels. BmKDfsin4 was recently identified at the protein level by our research group [42]. The four defensins (BmKDfsin2, BmKDfsin3, BmKDfsin4 and BmKDfsin6) were classified into the invertebrate CSαβ defensin family based on their genomic organization, predicted amino acid sequences and phylogenetic analysis. Using whole-scorpion bacterial infection model, sequencing of the potential promoter region and a dual luciferase promoter activity assay, we concluded that BmKDfsin3, BmKDfsin4 and BmKDfsin6 are constitutively expressed at the mRNA level, which was consistent with the expression of previously investigated scorpion defensins. The defensins LqDef [27] and AaDef [28] from the scorpions *L. quinquestriatus* and *A. australis* respectively, are both constitutively expressed independent of immune stimulus. Another scorpion defensin, Cll-dlp [30], exhibits an inducible liberation of stored peptides following CON. Additionally, some scorpion defensin-like peptides, such as scorpine [43], opiscorpines 1–4 [36] and Heteroscorpine-1 [44], exhibit no response to immune stimuli and have identical patterns of constitutive expression. All these data suggest that most defensins or defensin-like genes from scorpions are constitutively expressed.

### Diversification and evolution of defensin expression patterns in invertebrates

Defensins are a large family of antimicrobial peptides that belong to a variety of categories and that exhibit highly complex expression patterns in response to immune challenge [45]. Vertebrate α-defensins are frequently confirmed to be constitutively expressed in paneth cells and neutrophils of humans, monkeys and rodents [18]. By contrast, almost all vertebrate β-defensins exhibit inducible expression, except HBD1, which has high levels of basal expression but can also be induced [18,46,47]. The expression patterns of invertebrate CSαβ defensins are most complex and include the following three types: CON independent of exogenous immune stimulus, IND in response to an immune challenge or inducible protein release from storage in haemocyte granules stimulated by immune challenge but without transcriptional regulation [30]. The two main expression patterns of vertebrate defensins and three types of invertebrate defensins belong to two types of expression at the transcriptional level: one is CON independent of exogenous immune stimulus and the other is IND in response to an immune challenge.

**Figure 7:**
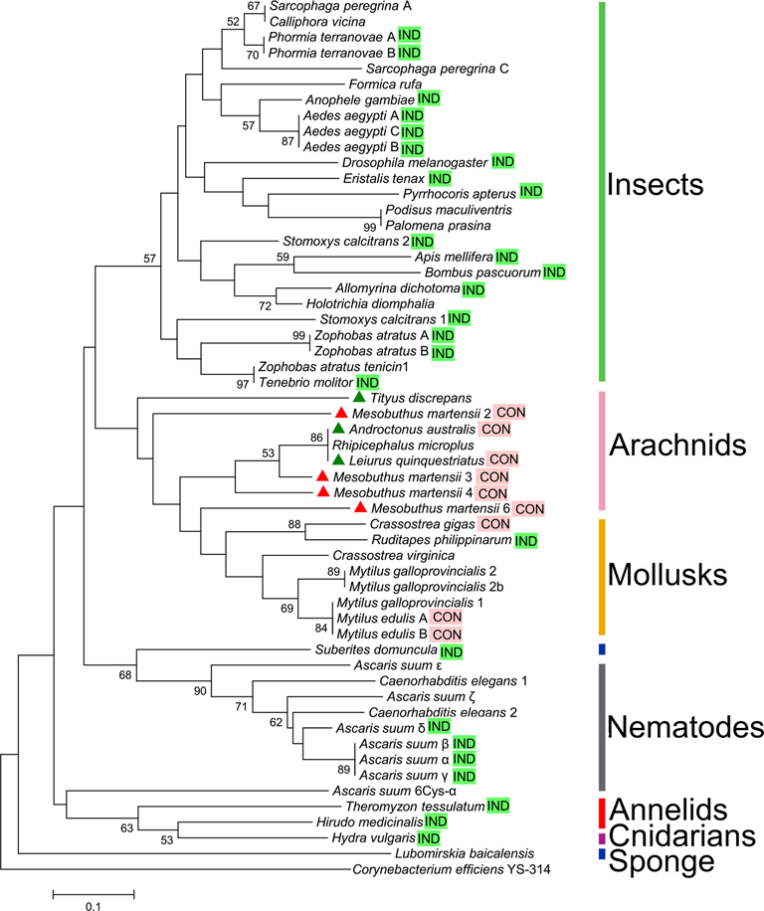
Diversification of CSαβ defensin expression patterns in invertebrates The phylogenetic tree was constructed using the Neighbor-Joining method in MEGA 5.1. Bootstrap sampling was reiterated 10000 times. Numbers above nodes represent bootstrap values greater than 50% (values below 50% not shown). The defensin of the bacterium *Corynebacterium efficiens* YS-314 was used as the outgroup. Evolutionary analysis using the Maximum-Parsimony method yielded a similar topology tree. The sequence information included in the analysis of the defensins is presented in Table 1 and Supplementary Table S2. CSαβ defensins with the expression pattern of IND are indicated with bright green background ‘IND’, while CSαβ defensins with the expression mode of CON are indicated with pink background ‘CON’. The four defensins from the scorpion *M. martensii* are highlighted with red triangles, while defensins from other scorpion species are indicated with green triangles.

In our study, we characterized the diversity and evolution of the expression patterns of defensin genes by analysing the most widespread group of defensins, the invertebrate CSαβ defensin family. As was shown in Table 1 and Figure 7, CSαβ defensin genes from insects, lower invertebrates (including nematodes, annelids, cnidarians and sponges), and some of arachnids and mollusks were found to be IND, whereas those from most of arachnids and mollusks are CON (Table 1 and Figure 7). The ancient species of invertebrates clearly had the IND type of CSαβ defensins.

**Table 1:** Typical invertebrate CSαβ defensin genes and their expression patterns

Abbreviated name and category	Species	Expression pattern*	Accession number
**Insects**			
AaeA	*Aedes aegypti*	IND	P91793
AaeB	*Aedes aegypti*	IND	AAD40114
AaeC	*Aedes aegypti*	IND	AAD40116
Adi	*Allomyrina dichotoma*	IND	Q10745
Aga	*Anopheles gambiae*	IND	Q17027
Ame1	*Apis mellifera*	IND	C55392
Bpa	*Bombus pascuorum*	IND	P81462
Dme	*Drosophila melanogaster*	IND	P36192
Ete	*Eristalis tenax*	IND	CAM92111
Orh	*Oryctes rhinoceros*	IND	O96049
Pap	*Pyrrhocoris apterus*	IND	P37364
PteA	*Phormia terraenovae*	IND	1ICA
PteB	*Phormia terraenovae*	IND	P10891
Sbu	*Sarcophaga bullata*	IND	1L4V
Sca1	*Stomoxys calcitrans*	IND	O16136
Sca2	*Stomoxys calcitrans*	IND	O16137
Tmo	*Tenebrio molitor*	IND	BAA04552
ZatA	*Zophobas atratus*	IND	AAB20745
ZatB	*Zophobas atratus*	IND	AAB20746
Ace	*Apis cerana*	NC	ACH96385
Ame2	*Apis mellifera*	NC	P17722
Big	*Bombus ignites*	NC	AAQ94318
Cvi	*Calliphora vicina*	NC	C0HJX7
Faq	*Formica aquilonia*	NC	AY875720
Fru	*Formica rufa*	NC	9672756
Hdi	*Holotrichia diomphalia*	NC	JC2554
HrhA	*Rhodnius prolixus*	NC	AAO74624
HrhB	*Rhodnius prolixus*	NC	AAO74625
HrhC	*Rhodnius prolixus*	NC	AAO74626
Mde1	*Mayetiola destructor*	NC	AAY82237
Ppr	*Palomena prasina*	NC	P80407
SpeA	*Sarcophaga peregrina*	NC	P18313
SpeC	*Sarcophaga peregrina*	NC	P31530
**Arachnids**			
Ahe1	*Amblyomma hebraeum*	IND	AY437137
Hlo2	*Haemaphysalis longicornis*	IND	ABO28925
Hlo3	*Haemaphysalis longicornis*	IND	ABO28926
OmoA	*Ornithodoros moubata*	IND	BAB41028
OmoB	*Ornithodoros moubata*	IND	BAB41027
OmoC	*Ornithodoros moubata*	IND	BAC10303
OmoD	*Ornithodoros moubata*	IND	BAC10304
Aau	*Androctonus australis*	CON	P56686
Bmi	*Boophilus microplus*	CON	AAO48943
Csa	*Cupiennius salei*	CON	
Cli	*Centruroides limpidus*	CON	P83738
Hlo1	*Haemaphysalis longicornis*	CON	AB105544
Ipe	*Ixodes persulcatus*	CON	BAH09304
Isc1	*Ixodes scapularis*	CON	AAV74387
Lqu	*Leiurus quinquestriatus*	CON	P41965
Mma2	*Mesobuthus martensii*	CON	
Mma3	*Mesobuthus martensii*	CON	
Mma4	*Mesobuthus martensii*	CON	
Mma6	*Mesobuthus martensii*	CON	
Ahe2	*Amblyomma hebraeum*	NC	AY437138
Asp	*Argiope spp*	NC	AAW01790
Dva	*Dermacentor variabilis*	NC	AAO24323
Iri1	*Ixodes ricinus*	NC	DQ361064
Iri2	*Ixodes ricinus*	NC	AY335442
Isc2	*Ixodes scapularis*	NC	EEC17844
Isc3	*Ixodes scapularis*	NC	EEC08554
Meu	*Mesobuthus eupeus*	NC	ABR21037
Mgi	*Mesobuthus gibbosus*	NC	CAL48845
Tdi	*Tityus discrepans*	NC	P0CF77
**Mollusks**			
Rph	*Ruditapes philippinarum*	IND	ALO24364
MedA	*Mytilus edulis*	CON	P81610
MedB	*Mytilus edulis*	CON	P81611
Cgi	*Crassostrea gigas*	NC	ACQ76287
Cvi	*Crassostrea virginica*	NC	P85008
Mga1	*Mytilus galloprovincialis*	NC	AAD45117
Mga2	*Mytilus galloprovincialis*	NC	AAD45118
Mga2b	*Mytilus galloprovincialis*	NC	AAD52660
**Nematodes**			
Asu-α	*Ascaris suum*	IND	BAA89497
Asu-β	*Ascaris suum*	IND	BAC00497
Asu-γ	*Ascaris suum*	IND	BAC00498
Asu-δ	*Ascaris suum*	IND	BAC00499
Asu-ε	*Ascaris suum*	NC	BAC41495
Asu-ζ	*Ascaris suum*	NC	BAC57992
Asu-6Cys-α	*Ascaris suum*	NC	AB086059
Cel1	*Caenorhabditis elegans*	NC	BAA89489
Cel2	*Caenorhabditis elegans*	NC	BAA89490
**Annelids**			
Hme	*Hirudo medicinalis*	IND	EU156754
Tte	*Theromyzon tessulatum*	IND	AY434032
**Cnidarians**			
Hvu	*Hydra vulgaris*	IND	B3RFR8
**Sponges**			
Sdo	*Suberites domuncula*	IND	CCC55928
Lba	*Lubomirskia baicalensis*	NC	CCC55929

* NC indicates not clear.

*CON indicates constitutive transcription

*IND indicates inducible transcription

### Evolutionary driving force and possible biological role of invertebrate CSαβ defensin expression types

Since CSαβ defensins from invertebrates have undergone unique routes of diversification and evolution along with the evolution of species from low to high, the driving force and potential biological role behind the evolution and diversification of the CSαβ defensin family are of interest. As shown in Figure 7, the IND type of CSαβ defensins is found to exist in all insects and lower invertebrates, and some mollusks and arachnids, whereas the CON expression type of CSαβ defensins occurs only in both mollusks and arachnids. In addition, CSαβ defensins of insects and arachnids exhibit significantly different features of molecular evolution. CSαβ defensins from the same species of insects (such as *Zophobas atratus, Phormia terraenovae* and *Aedes aegypti*) cluster together and share the same expression type with each other, suggesting that the CSαβ defensin genes in these species are the product of a recent gene duplication event and that the expression type was established before this event. Most of the CSαβ defensins arising from gene duplication exhibit IND expression. In species other than insects, CSαβ defensins from the same species (such as the scorpion *M. martensii* and the tick *Haemaphysalis longicornis*) do not cluster together, but most have the same expression type as arachnids, which implies that CSαβ defensin genes from these species underwent gene duplication before the split of scorpions, spiders and ticks and that the CON expression type of CSαβ defensins was formed after the gene duplication. This result was also consistent with the occurrence of CSαβ defensin CON expression type in mollusks. The use of the same CSαβ defensin expression mode within species and between closely genetically related species implies that the driving force behind the evolution of CSαβ defensin expression modes in invertebrates may be the similar immune challenges faced by these species. Most mollusk and arachnid species, such as snails and scorpions, usually live in an environment rich of a wide variety of microbial populations. Evolutionary CON expression type of CSαβ defensin genes in mollusks and arachnids possibly help them to defend microbial infection.

## Supporting information

**Fig S1.** PCR validation of defensin genes from the scorpion *M. martensii* The genomic DNA of the scorpion *M. martensii* was used as the PCR template. M, 1 Kb DNA Ladder. 1, BmKDfsin1. 2, BmKDfsin2. 3, BmKDfsin3. 4, BmKDfsin4. 5, BmKDfsin5. 6, BmKDfsin6.

**Fig S2.** PCR amplification of promoter regions of three defensin genes the scorpion *M. martensii* Three defensins (BmKDfsin3, BmKDfsin4 and BmKDfsin6) from the scorpion *M. martensii* were selected to clone their promoter regions. M, 1Kb DNA Ladder. 1, BmKDfsin3. 2, BmKDfsin4. 3, BmKDfsin6.

**Table S1.** PCR primers for the validation of potential defensin genes

**Table S2.** Amino acid sequence information of defensins used in Figure 3 and Figure 7

**Table S3.** Real-time PCR primers for *M. martensii* defensin and actin genes

**Table S4.** Primers for cloning the promoter regions of defensin genes

**Table S5.** Primers for inserting the promoter regions of into pGL3-Basic vector

**Table S6.** Six potential defensin genes characterized from the scorpion *M. martensii* genome

## Abbreviations

CCTCC: China Center for Type Culture Collection
CON: constitutive transcription
CSαβ: cysteine-stabilized α-helix/β-sheet motif defensin
HEK293T: human embryonic kidney
HNF-5: hepatic nuclear factor 5
ICRE: interferon consensus response element
IND: inducible transcription
LPS: lipopolysaccharide
LTA: lipoteichoic acid
NF-ELAM1: nuclear factor endothelial leucocyte adhesion molecule 1
NF-IL6: nuclear factor interleukin 6
