# Supplementary material for: Molecular characterization and expression analysis of CSαβ defensin genes from the scorpion Mesobuthus martensii
Source: Biosci Rep. 2017 Dec 15;37(6):BSR20171282. doi: 10.1042/BSR20171282 (PMC6435467; doi:10.1042/BSR20171282)

Supplementary information

Molecular characterization and expression analysis of CSαβ defensin genes from the scorpion *Mesobuthus martensii*

Yange Lang[a], Xiaohuan Pi[b], Zhiyong Di[a,c], Qian Zhang[a], Huijuan Wang[a], Bingzheng Shen[a,d], Fangfang Li[a], Gaomin Liu[a], Yao Yu[e], Xuan Li[e], Yingliang Wu[a], Wenxin Li[a], Zhijian Cao[a,*]

[a]State Key Laboratory of Virology, College of Life Sciences, Wuhan University, Wuhan 430072, China
[b]College of Basic Medicine, Lanzhou University, Lanzhou 730000, China
[c]College of Life Sciences, University of Science and Technology of China, Hefei 230022, China
[d]Department of Pharmacy, Renmin Hospital, Wuhan University, Hubei, 430060, China
[e]Key Laboratory of Synthetic Biology, Institute of Plant Physiology and Ecology, Shanghai Institutes for Biological Sciences, Chinese Academy of Sciences, Shanghai 200031, China

*Author to whom correspondence should be addressed. E-mail: zjcao@whu.edu.cn. Tel: ++86-27-68752831. Fax: ++86-27-68756746.

Supplementary tables

Table S1. PCR primers for the validation of potential defensin genes

Primer names	Sequences
	Forward primers
BmKDfsin1-FP	5'-TGCAGATAAATTATGCTG-3'
BmKDfsin2-FP	5'-TTTTTGACAATACAGTTGACTCCTA-3'
BmKDfsin3-FP	5'-GGCTCGCCCATTGAACCCATAAACC-3'
BmKDfsin4-FP	5'-ATCCGGGTCCCACACATCCAGATTT-3'
BmKDfsin5-FP	5'-ATACTACGTCCGTGTCCG-3'
BmKDfsin6-FP	5'-TGAAATCAGTTTTGCAGAATTGCCA-3'
	Reverse primers
BmKDfsin1-RP	5'-TTCTAGCAATGATGAAGT-3'
BmKDfsin2-RP	5'-CGAAGAGACAGGGAAAGGAAAGACT-3'
BmKDfsin3-RP	5'-ATGTCCAGATCAAAATGGTTCCGTC-3'
BmKDfsin4-RP	5'-ATGAAACCACCACATGAGCCGAAAG-3'
BmKDfsin5-RP	5'-GGCAATCATTGCAGTCAA-3'
BmKDfsin6-RP	5'-AGATGTGCGGTAAAACCACCTAAAG-3'

Table S2. Amino acid sequence information of defensins used in Figure 3 and Figure 7

Names	Sequences
Homo_sapiens_1	VSLAWDESLAPKHPGSRKNMACYCRIPACIAGERRYGTCIYQGRLWAFCC
Homo_sapiens_3	VSLAWDESLAPKHPGSRKNMDCYCRIPACIAGERRYGTCIYQGRLWAFCC
Homo_sapiens_4	ISFAWDKSSALQVSGSTRGMVCSCRLVFCRRTELRVGNCLIGGVSFTYCC
Macaca_mulatta	VSLAWDESLAPKDSVPGLRKNMACYCRIPACLAGERRYGTCFYLGRVWA FCC
Oryctolagus_cuniculus	IYVKEHESSALEALGVKAGVVCACRRALCLPRERRAGFCRIRGRIHPLCC
Rattus_norvegicus_1	ISFGGDKGTALQDADVKAGVTCYCRSTRCGFRERLSGACGYRGRIYRLCC
Mus_musculus	VSFGDPEGTSLQEESLRDLVCYCRSRGCKGRERMNGTCRKGHLLYTLCC
Cavia_porcellus	ISFWEEESTSLEDAGAGAGRRCICTTRTCRFPYRRLGTCIFQNRVYTFCC
*Homo_sapiens*_HBD2	CLKSGAICHPVFCPRRYKQIGTCGLPGTKCC
*Homo_sapiens*_HBD3	YYCRVRGGRCAVLSCLPKEEQIGKCSTRGRKCC
Bos_taurus	CRWNMGVCIPISCPGNMRQIGTCFGPRVPCC
Bubalus_bubalis	CRRNRGFCLAFWCPGSMRQIGTCFGFPVKCC
Capra_hircus	CHRIKGVCAPDRCPRNMRQIGTCFGPPVKCC
Rattus_norvegicus_2	CLTKGGVCWGPCTGGFRQIGTCGLPRVRCC
Meleagris_gallopavo	CRIRGGFCRFGSCRFPHIAIAKCATFIPCC
Gallus_gallus	MQCGYRGTFCTPGKCPYGNAYLGLCRPKYSCC
Paralichthys_olivaceus	LDCSTIQGVCKDSCLSTEFSIGALGCSAESSTVCC
Takifugu_rubripes	WTCPSLSGVCRKVCLPTEMFFGPLGCGKGFQCC
Tetraodon_nigroviridis	WACPSLNGVCRKVCLPTELFFGPLGCGKGFLCC
Oncorhynchus_mykiss	FSCPTLSGVCRKLCLPTEMFFGPLGCGKGFLCC
Panulirus_japonicus_1	NDCGSNGGSCTRGYCSYSNRLPYTCSLGRTCC
Panulirus_japonicus_2	LDCRTNGGRCVTGYCSNTLPYSCGGGAICC
Branchiostoma_floridae	HSCANNRGWCRSSCFSHEYIDYYNSAVCGRYRCC
Branchiostoma_belcheri_tsingtauense	HSCANNRGWCRSRCFSHEYIDSWHSDVCGSYDCC
Argopecten_irradians	HSCYGNRGWCRSSCRSYEREYRGGNLGVCGSYKCC
Ruditapes_philippinarum	RRCLSGRGFCRAICSIFEEPVRGNIDCYFGYNCC
Crassostrea_gigasbig_1	HSCANNRGWCRPTCFSHEYTDWFNNDVCGSYRCC
Crassostrea_gigasbig_2	HSCANNRGWCRPTCYSYEYTDWFNNDVCGSYRCC
Crassostrea_gigasbig_3	HSCANNRGWCRESCFSHEYTDWANTFGVCGSYFCC
Tachypleus_tridentatus	HSCAGNRGWCRSKCFRHEYVDTYYSAVCGRYFCC
Mesobuthus_eupeus	KYCSEDPLECNEHCKTKNQIGVCHGANGKEKCSC
Centruroides_limpidus	GACQFWSCNSSCISRGYRQGYCWGIQYKYCQC

Table S2. Amino acid sequence information of defensins used in Figure 3 and Figure 7 (continued)

Names	Sequences
Androctonus_australis	FGCPFNQGACHRHCRSIRRRGGYCAGLFKQTCTC
Leiurus_quinquestriatus	FGCPLNQGACHRHCRSIRRRGGYCAGFFKQTCTC
*Mesobuthus_martensii*_2	YGCPVNEKICQEHCRSIGRSGGNCTGFRRKDCVC
*Mesobuthus_martensii*_3	FGCPFNQGKCHRHCRSIRRRGGYCDGFLKQRCVC
*Mesobuthus_martensii*_4	FGCPFNQGQCHKHCQSIRRRGGYCDGFLKTRCVC
*Mesobuthus_martensii*_6	FGCPLFQFACDSHCRGMGRKGGYCGGNFKLTCIC
Tityus_discrepans	RYCPRNPEACYNYCLRTGRPGGYCGGRSRITCFC
Dermacentor_variabilis	FGCPLNQGACHNHCRSIRRRGGYCSGIIKQTCTC
*Mytilus_galloprovinsialis*_1	FGCPNNYQCHRHCKSIPGRCGGYCGGWHRLRCTC
*Mytilus_galloprovincialis*_2	FGCPNNYACHQHCKSIRGYCGGYCASWFRLRCTC
*Mytilus_edulis*_A	FGCPNDYPCHRHCKSIPGRXGGYCGGXHRLRCTC
*Mytilus_edulis*_B	FGCPNDYPCHRHCKSIPGRYGGYCGGXHRLRCTC
*Crassostrea_gigas*_1	FGCPRDQYKCNSHCQSIGCRAGYCDAVTLWLRCTC
*Crassostrea_gigas*_2	FGCPGDQYECNRHCRSIGCRAGYCDAVTLWLRCTC
*Crassostrea_gigas*_m	FGCPGNQLKCNNHCKSISCRAGYCDAATLWLRCTC
Crassostrea_virginica	FGCPWNRYQCHSHCRSIGRLGGYCAGSLRLTCTC
Haliotis_discus_discus	VTCDLLSLQIMGNSFGDSACAAHCIGLHHSGGHCSGGVCVC
Haliotis_discus_hannai	VTCDLLSFQIGGFSFGDSACAAHCIVLHHNGGHCSNGVCVC
Anopheles_gambiae	ATCDLASGFGVGSSLCAAHCIARRYRGGYCNSKAVCVC
Aedes_aegypti	ATCDLLSGFGVGDSACAAHCIARRNRGGYCNAKKVCVC
Drosophila_melanogaster	ATCDLLSKWNWNHTACAGHCIAKGFKGGYCNDKAVCVC
Pseudoplectania_nigrella	FGCNGPWDEDDMQCHNHCKSIKGYKGGYCAKGGFVCKC

The six conserved cysteine residues which forms the disulfide bonds are highlighted with an orange color.

Table S3. Real-time PCR primers for *M. martensii* defensin and actin genes

Primer names	Sequences
	Forward primers
Q-BmKDfsin3-FP	5'-CACTCTTGAAATGGGAATGG-3'
Q-BmKDfsin4-FP	5'-CTTGAAATGGGAATAGTGG-3'
Q-BmKDfsin6-FP	5'-CTATGGAGATTACGATGG-3'
Q-β-actin-FP	5'-GGTATAGTGACAAATTGGGATG-3'
	Reverse primers
Q-BmKDfsin3-RP	5'-CATCGCAATATCCTCCTCTT-3'
Q-BmKDfsin3-RP	5'-ATCGCAATATCCTCCTCTT-3'
Q-BmKDfsin3-RP	5'-AATACCCTCCTTTTCGTC-3'
Q-β-actin-RP	5'-TTGCCTTAGGATTCAGTGGG-3'

Table S4. Primers for cloning the promoter regions of defensin genes

Primer names	Sequences
	Forward primers
p-BmKDfsin3-FP	5’-GCACACAAATGTAAGG-3’
p-BmKDfsin4-FP	5’-GCCCTTCGACAGGTTTC -3’
p-BmKDfsin6-FP	5’-GATTTGTTTTTGTAGTCTATGACAT-3’
	Reverse primers
p-BmKDfsin3-RP	5’-GCTTCCACCATTCCCATTTC-3’
p-BmKDfsin4-RP	5’-GCTTCCACTATTCCCATTTCT-3’
p-BmKDfsin6-RP	5’-CGAATCCAGCTTCTACCATCG-3’

Table S5. Primers for inserting the promoter regions of into pGL3-Basic vector

Primer names	Sequences
	Forward primers
pGL3-BmKDfsin3-FP	5’-CGACGCGTGCACACAAATGTAAGG-3’
pGL3-BmKDfsin4-FP	5’-CGACGCGTGCCCTTCGACAGGTTT-3’
pGL3-BmKDfsin6-FP	5’-CGACGCGTGATTTGTTTTTGTAGT-3’
	Reverse primers
pGL3-BmKDfsin3-RP	5’GAAGATCTGCTTCCACCATTCCCATTTC-3’
pGL3-BmKDfsin4-RP	5’-CCGAGATCTGCTTCCACTATTCCCATTT-3’
pGL3-BmKDfsin6-RP	5’-CCGCTCGAGCGAATCCAGCTTCTACCAT-3’

Table S6. Six potential defensin genes characterized from the scorpion *M. martensii* genome

Scallfold ID	E-value	Score	Transcript	Name
NODE_7320178_length_147024_cov_27.792116_9_split_1_split2	6.00E-18	78.2	comp1050_c0_seq1 len=467	BmKDfsin5
NODE_7421102_length_247998_cov_28.593920_3_split_0_split2	7.00E-14	66.2	comp92_c0_seq1 len=1607	BmKDfsin3
NODE_7421102_length_247998_cov_28.593920_2_split_9_split2	1.00E-12	63.2	comp92_c0_seq5 len=232	BmKDfsin4
NODE_7796716_length_1260638_cov_29.621883_29_split_1_split2	6.00E-10	54.3	comp14_c1_seq1 len=251	BmKDfsin6
NODE_4521545_length_29012_cov_18.969770_0_split	4.00E-07	47	None	BmKDfsin1
NODE_4521545_length_29012_cov_18.969770_1_split	5.00E-07	47	comp12795_c0_seq4 len=146	BmKDfsin2

Supplementary figure legends

Fig S1. PCR validation of defensin genes from the scorpion *M. martensii*

The genomic DNA of the scorpion *M. martensii* was used as the PCR template. M, 1 Kb DNA Ladder. 1, BmKDfsin1. 2, BmKDfsin2. 3, BmKDfsin3. 4, BmKDfsin4. 5, BmKDfsin5. 6, BmKDfsin6.

Fig S2. PCR amplification of promoter regions of three defensin genes from the scorpion *M. martensii*

Three defensins (BmKDfsin3, BmKDfsin4 and BmKDfsin6) from the scorpion *M. martensii* were selected to clone their promoter regions. M, 1Kb DNA Ladder. 1, BmKDfsin3. 2, BmKDfsin4. 3, BmKDfsin6.

Fig S1

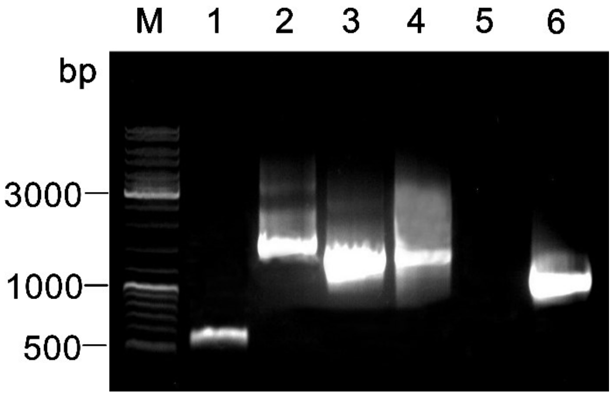

Fig S2

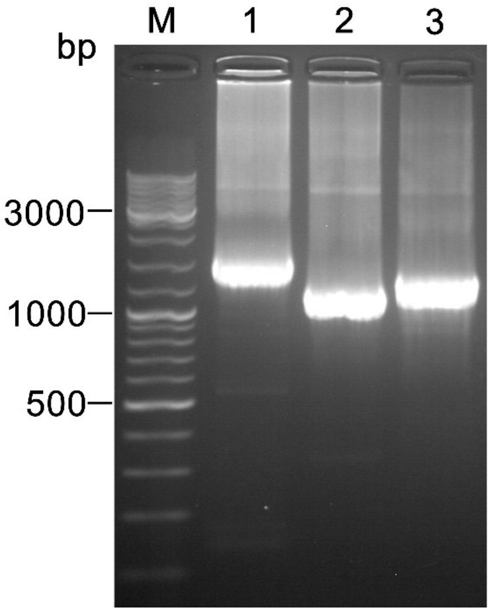

Supplement: Supplementary file 1 [file bsr20171282_Supp1.pdf]
